# IL-6/STAT3 signaling in prostate cancer: CAF-driven immune evasion and therapeutic opportunities

**DOI:** 10.3389/fimmu.2025.1736606

**Published:** 2026-01-15

**Authors:** Tao Zhou, Yuqi Li, Zhiyu Liu, Zhiqiang Zeng, Tao Li, Li Chen, Huan Zhao, Xiaochun Wu, Yuxuan Shen, Haitao Fan, Xiaoxiao Zhu, Yubo Zhou, Lunhong Zou, Dan Zhao

**Affiliations:** 1 Department of Urology, Santai Hospital Affiliated to North Sichuan Medical College, Mianyang, Sichuan, China; 2 Department of Urology, Affiliated Hospital of Southwest Medical University, Luzhou, Sichuan, China; 3 Nursing Department, Santai Hospital Affiliated to North Sichuan Medical College, Mianyang, Sichuan, China

**Keywords:** cancer-associated fibroblasts, interleukin-6, prostate cancer, therapeutic resistance, tumor microenvironment

## Abstract

Interleukin-6 (IL-6) plays a pivotal regulatory role in prostate cancer progression, contributing to therapy resistance and reshaping of the tumor microenvironment. This review outlines the clinical relevance of IL-6 as a potential prognostic biomarker and describes its mechanistic involvement in the development of castration resistance, with emphasis on its interplay with distinct cancer-associated fibroblast (CAF) subtypes. Elevated serum IL-6 levels in metastatic castration-resistant prostate cancer are associated with poor responses to docetaxel, enzalutamide, or abiraterone, and correlate with worse prognosis. Mechanistically, IL-6 promotes neuroendocrine differentiation and sustains cell survival under therapeutic stress through activation of signal transducer and activator of transcription 3 (STAT3), mitogen-activated protein kinase (MAPK), and androgen receptor signaling pathways. Recent single-cell studies reveal that prostate CAFs are highly heterogeneous. Certain subtypes are linked to extracellular matrix remodeling and fibrosis, while others exhibit inflammatory or immune-modulatory characteristics, differentially influencing tumor evolution. Specific CAF subsets have been strongly implicated in promoting castration resistance and adverse outcomes. Therapeutic strategies targeting the IL-6/IL-6R axis—such as neutralizing antibodies, advanced chimeric antigen receptor (CAR)-T designs, and combination regimens—are under active investigation. Simultaneously, modulating CAF plasticity to convert tumor-promoting phenotypes into tumor-restraining ones represents a promising therapeutic avenue. A deeper understanding of IL-6 functions across CAF subtypes may unlock novel precision therapy opportunities for prostate cancer.

## 1 Introduction

Prostate cancer is a major malignancy and a significant threat to men’s health worldwide (1, 2). When prostate cancer enters the castration-resistant phase, treating it becomes much harder, and this is a problem often encountered in clinics (3). Recently, the tumor microenvironment has been discussed more frequently because it affects how the disease develops and how patients respond to therapy. Among the many components involved, interleukin-6 (IL-6) and cancer-associated fibroblasts (CAFs) are two that have drawn particular interest (4–6).

A clinical study showed that metastatic castration-resistant prostate cancer (mCRPC) in patients with elevated levels of IL-6 usually indicates poor therapeutic effect (7). This cytokine not only can independently predict the response to chemotherapy (8), but also has a significant positive correlation with tumor burden and the extent of bone metastasis, which provides an important reference for the evaluation of advanced disease (9, 10).

IL-6 can act through several pathways inside the cell. For example, it induces signal transducer and activator of transcription 3 (STAT3) phosphorylation and activates mitogen-activated protein kinase (MAPK) signaling, and it can also influence androgen receptor transactivation (11). IL-6 induces neuroendocrine differentiation of tumor cells via the AMP−activated protein kinase (AMPK)/sirtuin 1 (SIRT1)/p38MAPK pathway, enabling survival in low-androgen conditions (12). Tumor cells in a long-term IL-6-rich environment will also gradually develop resistance to chemotherapy and hormone therapy through continuous activation of STAT3 (13) and inhibition of p53 function (14).

With the advancement of single-cell technology, researchers have found that CAFs in prostate cancer are significantly heterogeneous (15, 16). These cells not only have different functions but even show opposite biological characteristics: CAFs responsible for extracellular matrix remodeling can accelerate tissue fibrosis, while CAFs associated with lymphocytes can promote immune cell infiltration and sometimes even show tumor-suppressive effects (17). Of particular concern is that some specific CAF subtypes are strongly associated with the development of castration resistance and poor clinical outcomes (18, 19).

The interaction between CAFs and tumor cells forms a cytokine-rich tumor microenvironment (5, 20). IL-6 derived from CAFs, for example, can activate the monoamine oxidase A/mammalian target of rapamycin/hypoxia-inducible factor-1α (MAOA/mTOR/HIF-1α) pathway inside tumor cells, increasing the expression of CXCR4 and IL-6 receptors, and eventually making the tumor more aggressive (21, 22). Clinical observations have shown that in patients receiving docetaxel or abiraterone treatment, when the baseline IL-6 level is high and T-cell counts decrease significantly, immunosuppression is usually more obvious. This also supports IL-6 as a possible predictive indicator (23).

Treatment strategies targeting IL-6 signaling are under active investigation. Beyond monoclonal antibodies that block IL-6 function (4, 24), IL-6 receptor inhibitors, novel chimeric antigen receptor (CAR)-T designs (25), and combination strategies concurrently targeting STAT3 and IL-6R have been explored (26). Furthermore, CAFs exhibit considerable plasticity. For instance, yes-associated protein 1 (YAP1) inhibition can shift tumor-promoting CAFs toward a tumor-restraining phenotype and enhance CD8^+^ T-cell infiltration (17, 27). Some studies also indicate that the epigenetic regulator KMT2D can influence IL-6 expression levels (22).

Even with these findings, there are still many unknowns regarding the IL-6–CAF relationship. Future work may need to establish a more stable CAF classification system to support clinical grouping (28) and consider combination therapy approaches that target both IL-6 signaling and stromal remodeling, especially for advanced prostate cancer.

## 2 Interleukin-6 signaling and tumor microenvironment in prostate cancer

**Figure 1:**
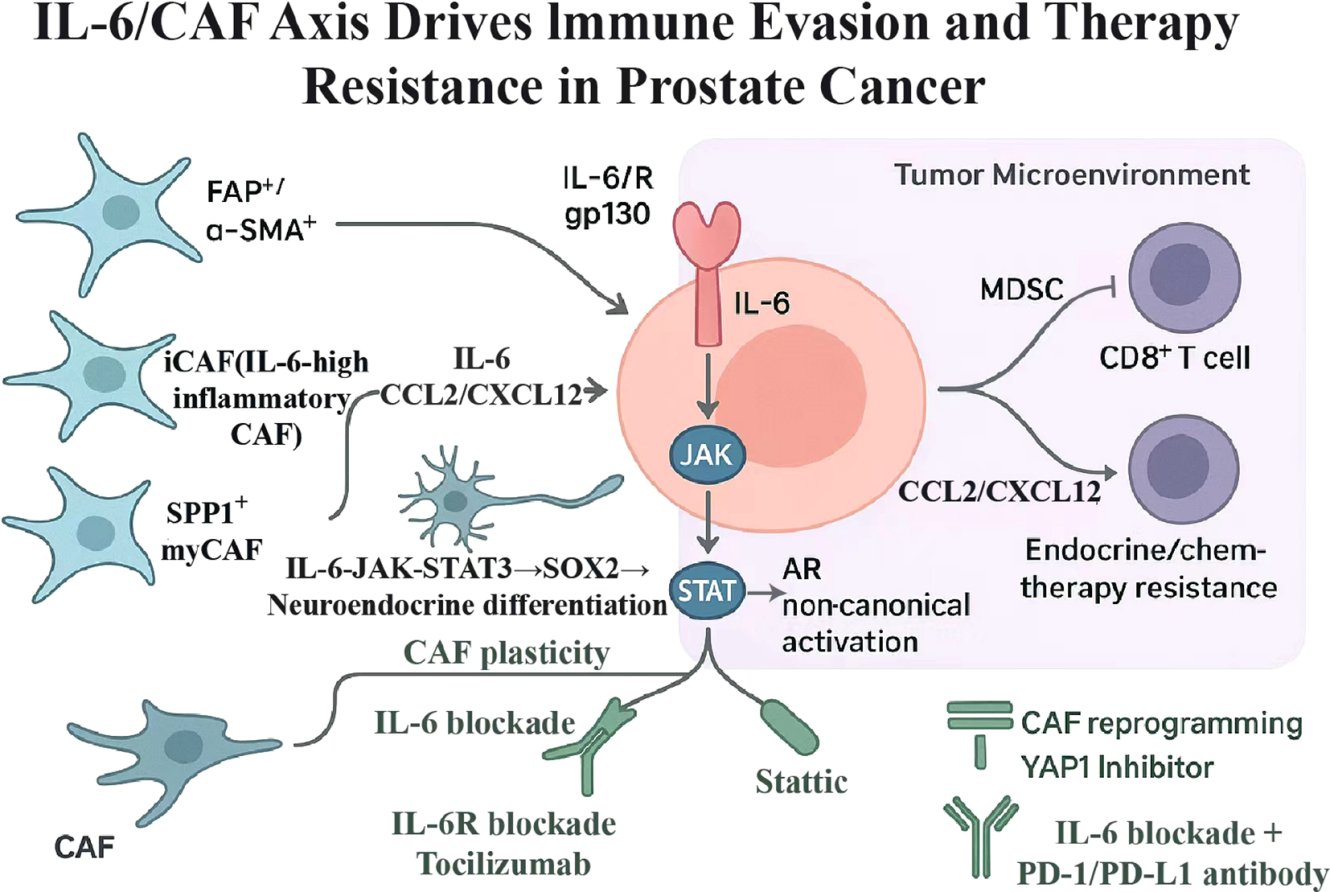
Mechanistic overview of IL-6/STAT3 signaling driving tumor progression and immune evasion in prostate cancer.

IL-6 is not only an inflammatory marker, but also a multifunctional factor involved in regulating tumor behavior during the development of prostate cancer (4, 29). It can act directly on cancer cells, and it may also affect tumor progression through changes in the surrounding microenvironment (**Figure 1**).

IL-6 can activate several oncogenic pathways at the cellular level, including STAT3, MAPK, and androgen receptor signaling (30, 31). The coordination of these pathways supports continuous tumor cell growth and survival. In LNCaP cells, IL-6 has been observed to promote neuroendocrine features via the AMPK/SIRT1/p38MAPK axis (12). It can also raise VCP expression, which may increase migratory and invasive behavior (32). Moreover, IL-6 may alter p53-mediated apoptosis through the JAK/STAT pathway, which could contribute to its role in chemoresistance (14, 30).

High IL-6 levels are frequently observed when systemic therapies such as docetaxel or enzalutamide begin to lose effectiveness (33, 34). Under these conditions, STAT3 can continue to sustain androgen receptor signaling even in the absence of adequate androgen (31). Treatment with tyrosine kinase inhibitors may also lead to a compensatory rise in IL-6, which can further intensify drug resistance (35). Some experimental studies have indicated that combining IL-6 inhibition with NF-κB blockade or metronomic treatment may enhance antitumor activity and help reduce resistance (8, 36).

Apart from its direct effects on tumor cells, IL-6 also influences the tumor microenvironment. IL-6 produced by CAFs can activate the MAOA/mTOR/HIF-1α pathway in nearby cancer cells, leading to increased receptor expression and promoting metastatic capacity (21).

High IL-6 levels in the microenvironment can also limit T-cell infiltration and disturb cytokine balance, making immune escape more likely (37, 38). In this way, tumor cells and CAFs reinforce each other and form an IL-6-driven loop that contributes to disease progression.

## 3 Therapeutic targeting and future perspectives

Therapeutic approaches that target IL-6 signaling have continued to make progress. Neutralizing antibodies, including siltuximab, can reduce IL-6-driven oncogenic activity and have shown acceptable safety in patients with prostate cancer (24, 39). In addition, several natural compounds have been reported to decrease IL-6 expression and restrict downstream signaling, in both androgen-sensitive and castration-resistant settings (40). Even so, the optimal dosing and the best way to combine these treatments are not yet fully settled and will require more study (4).

In addition to targeting cytokines, there is growing interest in regulating CAF plasticity as well. Blocking YAP1 signaling can change tumor-supporting CAFs into more tumor-restraining types and can also promote CD8^+^ T-cell infiltration, improving PD-1 therapy responses (17). Under androgen deprivation, SPP1^+^ myofibroblast-like CAFs may form, which are strongly related to treatment resistance, implying that modifying matrix remodeling might help enhance treatment outcomes (27, 41). In addition, KMT2D, as an upstream epigenetic regulator, can lower IL-6 transcription and thereby slow IL-6-driven tumor progression (22).

It is expected that combining IL-6/STAT3 pathway targeting with approaches that reshape stromal and immune components could help improve treatment outcomes. The integration of single-cell sequencing and spatial transcriptomics will help identify IL-6-responsive cell subtypes more precisely, supporting individualized treatment (42). Overall, co-targeting IL-6 and CAF-mediated signaling networks is likely to provide a new route to overcoming drug resistance and improving long-term outcomes in prostate cancer.

## 4 Single-cell analysis reveals the heterogeneity characteristics of cancer-associated fibroblasts in prostate cancer

Single-cell RNA sequencing has shown that the CAF population in prostate cancer is highly heterogeneous, which challenges the earlier assumption that CAFs form a relatively uniform cell group (19). Chen et al. reported that stromal and immune cells in prostate tumors display notable transcriptomic differences, especially in castration-resistant tumors, where certain endothelial and fibroblast subsets are strongly activated (15). The Song group, by integrating several single-cell datasets, identified two major CAF clusters: one mainly participates in extracellular matrix remodeling and contributes to invasion, while the other is linked to immune regulation and promotes CD8^+^ T-cell infiltration (17).

### 4.1 Molecular typing system of CAF subtypes

Researchers have proposed multiple classification frameworks to capture CAF heterogeneity. Pan et al. defined two main CAF subtypes—αSMA^+^CAV1^+^ CAFs-C0 and FN1^+^FAP^+^ CAFs-C1—with the FN1^+^FAP^+^ population showing a stronger association with castration resistance and adverse clinical outcomes (18). Building on this, Liu and colleagues expanded CAF taxonomy into three functionally distinct groups: myofibroblast-like CAFs, inflammatory/immune CAFs, and antigen-presenting CAFs, each contributing differently to prostate cancer progression (42). More recently, Ding et al. delineated four fibroblast populations (C0 IER2^+^, C1 ABCA8^+^, C2 ABI3BP^+^, and C3 MEOX2^+^), highlighting the ABCA8^+^ subtype as highly proliferative and potentially important for tumor growth and dissemination (43).

### 4.2 Functional specificity of cancer-associated fibroblast populations

Different CAF subtypes exert distinct biological effects. Zhang’s group identified an HSD17B2^+^ CAF subtype that accelerates tumor migration and promotes drug resistance through the AR/ITGBL1 signaling axis (44). Vickman et al. found that some CAF populations secrete high levels of CCL2 and CXCL12, enhancing myeloid cell infiltration and shaping an inflammatory microenvironment (19). A set of FAP^+^ fibroblasts has also been found to interact with SPP1^+^ macrophages through CSF1/CSF1R and CXCL/ACKR1 signaling. Through this interaction, they help build an immune-suppressive microenvironment (45).

### 4.3 Therapeutic significance and CAF plasticity

CAF plasticity—the capacity of fibroblasts to transition between distinct functional states—has important clinical implications. Song and colleagues showed that inhibiting YAP1 can reprogram CAFs from a tumor-promoting to a tumor-restraining phenotype, accompanied by increased CD8^+^ T-cell infiltration and improved responsiveness to PD-1 blockade (17). Li et al. suggested that androgen deprivation therapy can induce the emergence of SPP1^+^ myofibroblast-like CAFs, which are associated with sustaining castration resistance (27). In addition, Cheng and coworkers reported that late-onset tumors are often enriched for inflammatory CAF populations linked to epithelial–mesenchymal transition and drug resistance (46).

### 4.4 Prognostic and predictive application value

The heterogeneous features of CAFs also offer opportunities for diagnostic and prognostic assessment. For instance, Gao and colleagues developed a CAF-based scoring system that can predict progression-free survival and indicate immunosuppressive tumor phenotypes (28). Building on this, Qian et al.’s study introduced a CAF-associated molecular classification that stratifies patients according to the risk of biochemical recurrence (47). Li’s group also identified a prognostic signature derived from CAF-related markers, which may be useful in estimating recurrence probability and assessing likely response to immunotherapy (48). Together, these findings highlight the prognostic relevance of stromal heterogeneity in prostate cancer.

## 5 IL-6 and cancer-associated fibroblasts in prostate cancer diagnosis

CAFs can facilitate prostate cancer invasion through the MAOA/mTOR/HIF-1α signaling pathway, which increases the expression of CXCR4 and IL-6 receptors on tumor cells. Curcumin has been reported to interfere with this pathway and reduce IL-6 receptor overexpression, thereby restricting epithelial–mesenchymal transition, suggesting a potential chemopreventive role (21). In mCRPC, patients who are naturally insensitive to abiraterone or enzalutamide generally present with higher baseline IL-6 levels than those who respond to treatment. This heightened IL-6 is often accompanied by reduced T-cell counts and altered cytokine patterns, supporting the use of IL-6 as an indicator of treatment response (23).

### 5.1 Mechanism of interaction between bone microenvironment

The bone microenvironment has a significant influence on metastatic prostate cancer progression (49, 50). In PC-3 cells, IL-6 can raise PTHrP expression, enhancing cell survival signals. Under cytotoxic stress, IL-6 can exert anti-apoptotic effects, while zoledronic acid and dexamethasone differentially modulate PTHrP expression in PC-3 cells (51). Differences in inflammatory markers are also seen between benign and malignant tissues. Benign prostatic hyperplasia is often associated with elevated inflammatory cytokines (e.g., IL-6 and IL-8), whereas prostate cancer has been linked to increased growth factor signaling such as FGF2 (52). These differences may help refine diagnostic classification.

### 5.2 Diagnostic and therapeutic significance

IL-6 contributes not only to inflammatory regulation but also to glycosylation changes in tumors. Epigenetic control of COSMC and the associated rise in Tn antigen levels point to an interaction between cytokine signaling and carbohydrate metabolism (53). These molecular features could be useful for developing biomarkers to monitor disease progression and to clarify how inflammation promotes malignancy. Moreover, nanoparticle-based formulations (e.g., mangiferin-functionalized gold nanoparticles) have shown immunomodulatory effects in prostate cancer models, including reductions in pro-tumor cytokines such as IL-6 (54). Assessing IL-6 together with CAF-related markers may therefore assist in precision diagnosis and personalized treatment planning in prostate cancer.

## 6 Translational and clinical implications of the IL-6/CAF axis in prostate cancer

The IL-6/CAF axis is increasingly recognized for its translational relevance, influencing both prognosis assessment and individualized treatment planning. Elevated circulating IL-6 is associated with poorer outcomes in castration-resistant prostate cancer and has been explored as a prognostic biomarker in clinical studies (55). Furthermore, baseline IL-6 and soluble IL-6 receptor (sIL-6R) levels have been associated with prostate cancer progression and metastasis, supporting their potential utility in risk stratification (7). In the context of androgen receptor pathway inhibitors (e.g., abiraterone/enzalutamide), inflammatory cytokine profiles including IL-6 have also been reported in patients with *de novo* resistance, suggesting possible predictive value (23).

In terms of therapy, strategies aimed at IL-6 and CAF-associated signaling are being considered as options to modify existing treatment approaches. Clinical testing of IL-6 pathway blockade has been conducted in chemotherapy-pretreated CRPC using the anti-IL-6 antibody siltuximab (CNTO328) (55). Pharmacologic modulation of CAFs through YAP1, FAP, or lysyl oxidase inhibition may also help normalize the extracellular matrix and improve drug delivery efficiency (27). Combining matrix-targeted strategies with androgen deprivation therapy may further delay the development of castration resistance.

Several newer technologies are contributing to clinical translation. Spatial transcriptomics together with multiplex imaging approaches can map cytokine programs and CAF subtype organization within tumor tissue, supporting more precise patient stratification for pathway-targeted treatments (56). In parallel, artificial intelligence (AI)-based radiomics and seromics models are being explored as non-invasive tools to infer IL-6 activity and guide individualized immunotherapy.

Translating IL-6/CAF biology into clinical application still faces several obstacles. Key needs include establishing longitudinal methods to monitor IL-6/CAF activity, developing combination treatment strategies that can adjust to evolving disease states, and incorporating computational prediction tools into clinical trial design. With progress in these areas, the IL-6/CAF axis has the potential to shift from a mechanistic observation to a practical component of precision oncology in prostate cancer.

## 7 Discussion

In prostate cancer, the interaction between IL-6 and CAFs contributes directly to disease progression and resistance to therapy. When IL-6 signaling remains active, tumors tend to advance toward castration-resistant and immune-evasive states (55, 57, 58). Clinically, patients with higher serum IL-6 levels are more likely to experience biochemical relapse, radiographic progression, and reduced overall survival, indicating that IL-6 functions as both a prognostic marker and a treatment target (58, 59).

IL-6 activates multiple signaling cascades at the molecular level, including STAT3, phosphoinositide 3-kinase/protein kinase B (PI3K/AKT), and MAPK, which together support proliferation, reduced apoptosis, and neuroendocrine features (60). When androgens are limited, IL-6 can also promote a non-classical mode of AR activation, helping to maintain AR signaling and cellular plasticity (61). In addition, IL-6–STAT3 signaling alters metabolic patterns by increasing glycolysis and tolerance to oxidative stress, which contributes to therapy resistance.

CAFs, which make up a large portion of the tumor stroma, can reinforce these oncogenic signals. Distinct CAF subtypes release IL-6, CCL2, and CXCL12 and recruit myeloid-derived suppressor cells through extracellular matrix remodeling, ultimately dampening anti-tumor immune responses (6, 19). IL-6 produced by CAFs can also establish a paracrine circuit that encourages tumor cells to develop their own autocrine IL-6 signaling, strengthening STAT3–AR cross-regulation and supporting stem-like properties (14, 62). Under treatment pressure, CAFs may undergo state transitions (CAF plasticity) that have been associated with immune escape and therapeutic resistance (41).

Therapeutically, targeting the IL-6/STAT3 pathway has become an area of interest. Although monoclonal antibodies such as siltuximab and tocilizumab exhibit good tolerability, monotherapy benefits are limited (24, 26). Combination strategies—such as IL-6 blockade with STAT3 inhibitors or immune checkpoint inhibitors—have shown synergistic effects in preclinical resistance models. Targeting stromal components (e.g., YAP1 inhibition or FAP-directed treatments) may reprogram CAFs toward tumor-restraining phenotypes, enhancing T-cell infiltration and immunotherapy response (41). However, translating these results into clinical benefit remains challenging, partly due to the context-dependent roles of IL-6 signaling (11).

Future research should take advantage of single-cell and spatial transcriptomic methods to define IL-6-responsive cell populations and integrate multi-omics approaches to elucidate IL-6-mediated metabolic, epigenetic, and immune coordination. AI-based modeling can help clarify the dynamic interactions among cytokines, stroma, and immunity, predict treatment responses, and guide rational combination therapy design. Longitudinal monitoring of IL-6 using liquid biopsy or exosome-derived markers may provide a non-invasive approach for assessing disease activity and treatment outcomes.
