# Correction: IL-6/STAT3 signaling in prostate cancer: CAF-driven immune evasion and therapeutic opportunities

**DOI:** 10.3389/fimmu.2026.1789441

**Published:** 2026-01-22

**Authors:** Frontiers Production Office

**Keywords:** cancer-associated fibroblasts, interleukin-6, prostate cancer, therapeutic resistance, tumor microenvironment

There was a mistake in **Figure 1** as published. In the PDF of the article, the revised version of the Figure was not included. However, it is correct on the webpage. The corrected **Figure 1** appears below.

**Figure 1:**
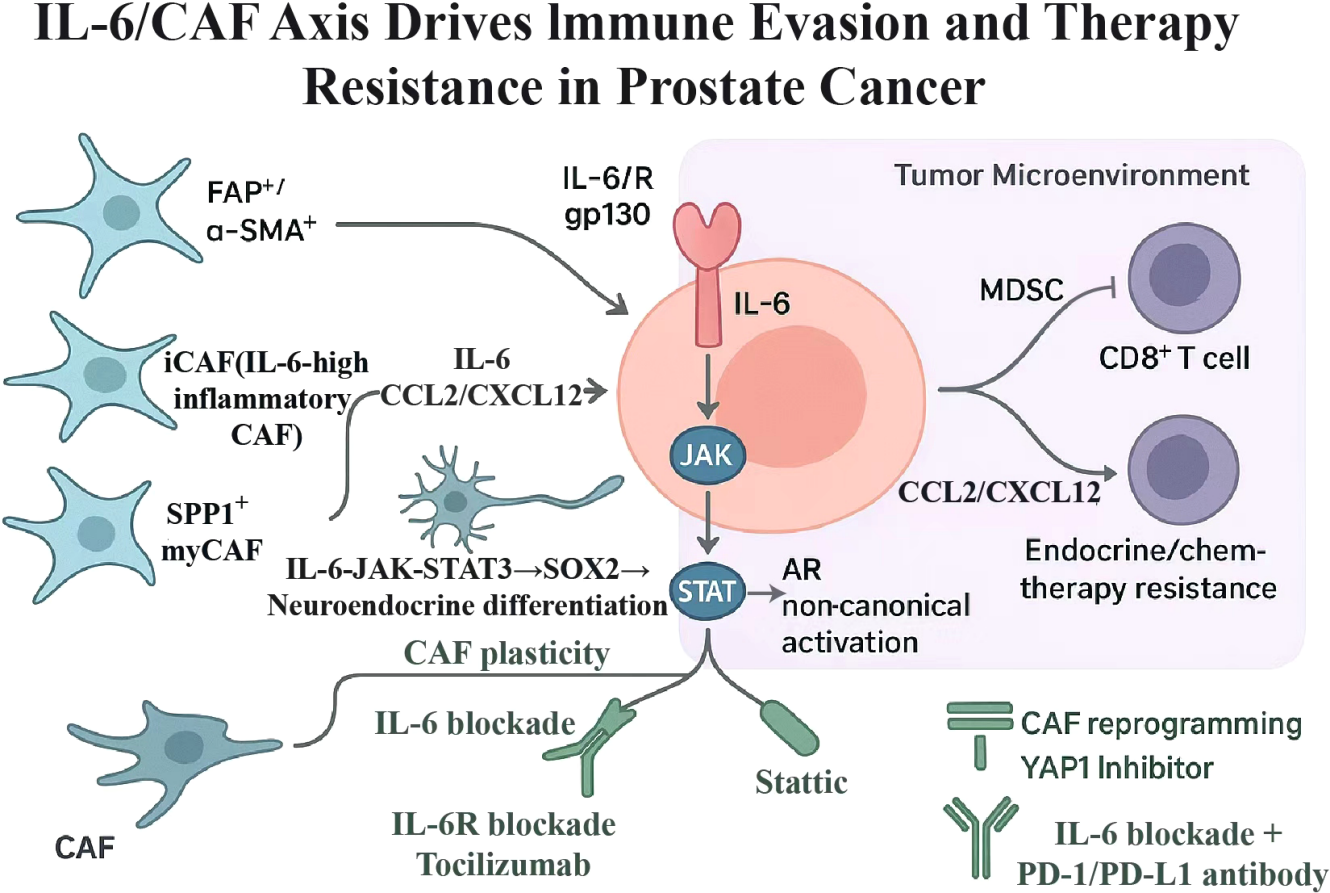
Mechanistic overview of IL-6/STAT3 signaling driving tumor progression and immune evasion in prostate cancer.

The original version of this article has been updated.

